# Editorial: Rising stars in nutrition and food science technology: innovative research on nutritional marine foods

**DOI:** 10.3389/fnut.2023.1206758

**Published:** 2023-05-17

**Authors:** Yueqi Wang, Yi Zhang, Yongqiang Zhao, Peng Liang

**Affiliations:** ^1^Key Laboratory of Aquatic Product Processing, Ministry of Agriculture and Rural Affairs, National R&D Center for Aquatic Product Processing, South China Sea Fisheries Research Institute, Chinese Academy of Fishery Sciences, Guangzhou, China; ^2^Co-Innovation Center of Jiangsu Marine Bio-industry Technology, Jiangsu Ocean University, Lianyungang, China; ^3^Key Laboratory of Efficient Utilization and Processing of Marine Fishery Resources of Hainan Province, Sanya Tropical Fisheries Research Institute, Sanya, China; ^4^Department of Food Science, The Pennsylvania State University, University Park, PA, United States; ^5^College of Food Science, Fujian Agriculture and Forestry University, Fuzhou, China

**Keywords:** marine food, nutritional functional components, flavor formation, food quality and safety, foodomics, freshness preservation

Marine foods is a kind of high quality food source for humans. With the increasing concern for human health, the demand for marine foods began to shift from the emphasis on survival and satiety to promote nutritional health and reduce the risk of morbidity needs. Marine foods is not only abundant in resources, but also rich in bioactive peptides, functional lipids, polysaccharides, vitamins and minerals and other healthy nutrition function factors, which is a health resources for human beings. Strengthening the basic research, applied basic research and key technological innovation of marine foods is of great significance to the high-value processing level of marine foods.

The current Research Topic aims at highlighting the progress and roles of scientific and technological innovations for the regulation of marine foods. In this collection, a total of eight papers have been published, related to the new technologies and discoveries on nutrition, flavor and quality improvement of marine foods, structural characterization and functional analysis of characteristic nutrients and nutritional functional components in marine foods, new ideas for controlling the quality of marine foods from the perspective of microbial molecular biology, as well as foodomics approach analysis for marine foods quality evaluation.

Modified atmosphere packaging (MAP) is an effective strategy for food preservation that inhibits microbial growth, decreases lipid oxidation, and protein degradation. Du et al. investigated the effects of sodium tripolyphosphate and trehalose soaking under vacuum permeating conditions on the physicochemical properties of shrimp (*Litopenaeus vannamei*) muscle with a focus on myofibrillar protein stability. The research showed the usefulness of sodium tripolyphosphate/trehalose combined with vacuum permeation treatments for improving the physicochemical properties of aquatic food products during frozen storage. Wang et al. evaluated effect of ε-polylysine (PL) and modified atmosphere packaging (MAP; 60% CO_2_/40% N_2_) on the bacterial community of greater amberjack (*Seriola dumerili*) filets and their physicochemical properties at 4°C. This investigation showed that the combination of PL and MAP has the potential to retain the quality and extend the shelf life of greater amberjack filets.

Flavor quality is one of the most important indicators of the intrinsic quality for marine foods, and is the primary condition for determining the acceptability and value of marine food products. Huang et al. evaluated the effect of atmospheric cold plasma (ACP) on snakehead (*Ophiocephalus argus* Cantor) surimi gels flavor at different treatment times using sensory evaluation and gas chromatography-ion mobility spectrometry (GC-IMS) analysis. This study provided new ideas for ACP application in the flavor characteristics of the snakehead surimi gels. Liao et al. identified volatile compounds in hairtail (*Trichiurus lepturus*) during the air-drying process using GC-IMS. The results showed that GC-IMS could detect volatile compounds in different air-dried hairtail rapidly and comprehensively.

It is well-known that marine foods are rich in lipids, especially n-3 polyunsaturated fatty acids, such as docosahexaenoic acid (DHA) and eicosapentaenoic acid (EPA). Sun et al. firstly determined the lipids species of *Crassostrea hongkongensis* fresh and dried products by the high performance liquid chromatography/quadrupole time-of-flight mass spectrometer (HPLC/Q-TOF-MS). Results showed that 1,523 lipid molecules were detected, in which polyunsaturated fatty acids mostly existed in such lipids as phosphoglyceride. In general, the results of this study are helpful to understand the lipids species comprehensively by HPLC/Q-TOF-MS compared with the current methods.

*Shewanella putrefaciens* is a special spoilage bacterium of marine foods during cold storage, which is easy to form biofilm and bring serious hazard to the marine foods quality. Xiong et al. compared the adhesion and biofilm formation of *S. putrefaciens* WS13 at 4°C with that at 30°C. Results showed that the swarming mobility of S. *putrefaciens* WS13 was weaker under 4°C, however, the adhesive force under 4°C was 4–5 times higher than that under 30°C. *S. putrefaciens* WS13 adapted to the cold stress by enhancing the expression of genes encoding diguanylate cyclases to promote bacterial adhesion and biofilm formation. This study provides a new idea to control marine foods quality from the perspective of microbial molecular biology.

The main symptoms of diabetes are hyperglycemia and insulin resistance. The inhibition of the starch digestion enzymes could effectively regulate starch digestion and glucose absorption, thereby slowing or treating the symptoms of postprandial hyperglycemia. Yin et al. used fucoxanthin as α-amylase inhibitor, and monitored the interactions of both biomolecules by using quartz crystal microbalance-admittance (QCM-A) instrument. It is helpful to clarify the mechanism of action of fucoxanthin on α-amylase, which further proved the potential of fucoxanthin to improve and treat postprandial hyperglycemia.

In recent years, the development of new marine foods resources with edible and medicinal value, as well as functional food development value has gradually received attention, including not only marine animals and plants, but also marine microorganisms. Yu et al. identified three groups of lipopeptides (surfactin, iturin, and fengycin) produced by *Bacillus velezensis* YA215 which isolated from sea mangroves at Beibu Gulf, Guangxi, China. Besides, one separation fraction named BVYA1 exhibits good antibacterial activity by disrupting the cell membrane of *Escherichia coli*. The results of this study are useful for further to screening a novel alternative to traditional antibiotics, and may have potential applications in food preservation and development of new biological bacterial inhibitors.

In conclusion, it can be inferred from these papers in this collection that the science and technology in marine foods processing has been rapidly developed. In the future research on marine foods should combine green and low carbon, focus on the nutritional functions of marine foods and develop different marine nutritional foods to better suit the consumption needs of different dietary groups in order to meet the needs of individualized nutrition.

## Author contributions

YW and PL wrote the editorial. YZhan and YZhao edited the editorial. All authors contributed to manuscript revision, read, and approved the submitted version.

